# Body Mass Index and Mortality in Non-Hispanic Black Adults in the NIH-AARP Diet and Health Study

**DOI:** 10.1371/journal.pone.0050091

**Published:** 2012-11-27

**Authors:** Yikyung Park, Patricia Hartge, Steven C. Moore, Cari M. Kitahara, Albert R. Hollenbeck, Amy Berrington de Gonzalez

**Affiliations:** 1 Division of Cancer Epidemiology and Genetics, National Cancer Institute, Rockville, Maryland, United States of America; 2 AARP, Washington, District of Columbia, United States of America; Virginia Commenwealth University, United States of America

## Abstract

**Background:**

Although the prevalence of obesity (body mass index, kg/m^2^, BMI ≥30) is higher in non-Hispanic blacks than in non-Hispanic whites, the relation of BMI to total mortality in non-Hispanic blacks is not well defined.

**Purpose:**

We investigated the association between BMI and total mortality in 16,471 non-Hispanic blacks in the NIH-AARP Diet and Health Study, a prospective cohort of adults aged 50–71 years.

**Methods:**

During an average of 13 years of follow-up, 2,609 deaths were identified using the Social Security Administration Death Master File and the National Death Index. Cox proportional hazard models were used to estimate relative risks and two-sided 95% confidence intervals (CI), adjusting for potential confounders.

**Results:**

Among individuals with no history of cancer or heart disease at baseline and had a BMI of 20 or greater, the relative risk for total death was 1.12 (95% CI:1.05, 1.19, for a 5-unit increase in BMI) in men and 1.09 (95% CI:1.03, 1.15) in women. Among never smokers with no history of cancer or heart disease at baseline, relative risks for total death for BMI 25–<30, 30–<35, 35–<40, and 40–50, compared with BMI 20–<25, were 1.27 (95% CI: 0.91, 1.78), 1.56 (95% CI: 1.07, 2.28), 2.48 (95% CI: 1.53, 4.05), and 2.80 (95% CI: 1.46, 5.39), respectively, in men and 0.78 (95% CI: 0.59, 1.04), 1.17 (95% CI: 0.88, 1.57), 1.35 (95% CI: 0.96, 1.90), and 1.93 (95% CI: 1.33, 2.81), respectively, in women.

**Conclusions:**

Our findings suggest that overweight is related to an increased risk of death in black men, but not in black women, while obesity is related to an increased risk of death in both black men and women. A large pooled analysis of existing studies is needed to systematically evaluate the association between a wide range of BMIs and total mortality in blacks.

## Introduction

The obesity epidemic in the US occurs in all racial/ethnic groups, but the prevalence of obesity (body mass index, kg/m^2^, BMI ≥30) varies by race. A report from the National Health and Nutrition Examination Survey 2007–2008 showed that non-Hispanic blacks had the highest prevalence of obesity (44%), followed by Hispanics (39%), and non-Hispanic whites (32%) in adults aged 20 years and older [1]. Among women aged 60 years and older, the prevalence of obesity was 51% in non-Hispanic blacks compared with 31% in non-Hispanic white women. In contrast, among men 60 years of age and older, the prevalence of obesity in non-Hispanic black men (38%) was the same as that in non-Hispanic white men.

The association between obesity and mortality has been well studied in whites. Two recent large pooled analyses of prospective cohort studies whose participants were predominantly white adults in North America and Western Europe found that overweight (BMI 25–<30) and obesity were associated with an increased risk of premature death [2],[3]. Another pooled analysis of Asians residing in Asia also reported that a high BMI was related to an increased risk of death in East Asians, but not in South Asians [4].

On the other hand, a limited number of studies, mostly small, examined the relation of BMI to total mortality in blacks and reported inconsistent results. Most previous studies observed a statistically non-significant positive association between obesity and total mortality in blacks [5], [6], [7], [8], [9], [10], [11]. This weak association may be partially due to small sample size and the inclusion of smokers or people with preexisting health conditions, which may have attenuated the association between BMI and total mortality, as shown in studies of whites [3], [9], [12]. A recent large study of blacks in the Southern Community Cohort found that obesity was not related to an increased risk of death among black men and women who were former or never smokers; this study even observed that the lowest risk of death was in people with BMI 30–<35 [13]. In contrast, another large study of black women, the Black Women’s Health Study, found that BMI was related to an increased risk of death when the analysis was restricted to never smokers [14]; the risk of death increased by 18% per 5-unit increase in BMI in healthy non-smoking women with BMI ≥20. The Multiethnic Cohort study also found that BMI was associated with an increased risk of total death among black men and women who never smoked [15].

In view of the high prevalence of obesity in blacks in the US, it is critical to define the relation of BMI to total mortality in this group. Therefore, we investigated the association between BMI and total mortality in 16,471 non-Hispanic blacks in the National Institute of Health (NIH)-AARP Diet and Health Study, a prospective cohort study of adults aged 50–71 years in the US.

## Subjects and Methods

### Study Population

NIH-AARP Diet and Health Study was initiated when AARP members aged 50–71 years from six states (California, Florida, Louisiana, New Jersey, North Carolina, and Pennsylvania) and two metropolitan areas (Atlanta, Georgia, and Detroit, Michigan) responded to a mailed questionnaire in 1995–96. Some states and metropolitan areas were selected to increase minority participants. Details of the NIH-AARP Diet and Health Study have been described previously [16].

Among 21,995 non-Hispanic black participants who returned the questionnaire with satisfactory data, we excluded individuals who indicated that they were proxies for the intended respondent (n = 395), those who did not report their height or body weight or had a BMI less than 15 or greater than 50 (n = 1,439), those who had any prevalent cancer except non-melanoma skin cancer or heart disease at baseline (n = 3,593), and those who were followed up for less than a year (n = 97). After exclusions, the analytic cohort consisted of 6,864 men and 9,607 women. The NIH-AARP Diet and Health study was approved by the Special Studies Institutional Review Board of the U.S. National Cancer Institute. All participants provided informed consent by virtue of completing and returning the questionnaire.

### Body Mass Index and Risk Factor Assessment

At baseline, we asked participants’ current height and body weight, which were used to calculate BMI. We also collected information on demographics, smoking status, number of cigarettes per day if ever smoked, time since quitting smoking, vigorous physical activity, some medical conditions, and menopausal hormone therapy use in women. Alcohol consumption was assessed using a food frequency questionnaire that was validated against two nonconsecutive 24-hour recalls [17].

### Mortality Ascertainment

We ascertained vital status through a periodic linkage of the cohort to the Social Security Administration Death Master File and follow-up searches of the National Death Index Plus for participants who matched to the Social Security Administration Death Master File, cancer registry linkage, questionnaire responses, and responses to other mailings. Our mortality ascertainment method was cost-effective and had more than 95% accuracy [18]. We used International Classification of Diseases, 9th and 10th revision to define death from cardiovascular diseases, cancer, and other causes except cardiovascular diseases, cancer, and accidents.

### Statistical Analysis

We used the Cox proportional hazards model to estimate relative risks (RRs) and two-sided 95% confidence intervals (CI), using age as the underlying time metric. The proportional hazard assumption, tested by modeling interaction terms of BMI with time, was upheld in the analyses. Participants were censored at the date of death or at the end of follow-up (December, 31, 2009 for total death and December, 31, 2008 for cause-specific deaths).

We categorized BMI into predefined groups: 15–<20, 20–<25 (reference category), 25–<30, 30–<35, 35–<40, and 40–≤50. We used BMI 20–<25 as a reference group for comparability to existing studies [13], [14]. We also estimated the risk of death per 5 unit increment in BMI among individuals with BMI ≥20, where the association between BMI and total mortality was linear.

The multivariate models adjusted for age, sex if men and women were combined, education, marital status, smoking status, including number of cigarettes per day and time since quitting smoking, physical activity, alcohol consumption, and menopausal hormone therapy use in women. For missing data in each covariate, we created an indicator variable. As previous studies have shown [3], [9], [12], controlling for smoking in the analysis of BMI in relation to total mortality may have residual confounding. Therefore, we also performed the analysis by restricting the study population to never smokers.

We evaluated whether the relation of BMI to total mortality is linear by comparing a nonparametric regression curve that used a restricted cubic spline with the linear model using the likelihood ratio test, and by visual inspection of the restricted cubic spline graphs [19], [20]. We also estimated the total mortality rate per 100,000 person-years, which was directly standardized to the sex-specific age distribution of the study population. All analyses were performed using SAS (version 9.1; SAS Institute, Inc, Cary, NC).

## Results

During an average 13 of years of follow-up for 16,471 black men and women, we identified 2,609 deaths (1,347 deaths in men and 1,262 deaths in women). The mean age at baseline was 61 years in both men and women (**Table 1**). The prevalence of class I (BMI: 30–<35), class II (BMI: 35–<40), and class III obesity (BMI ≥40) was 21%, 5%, and 2%, respectively, in men and 23%, 10%, and 5%, respectively, in women. Compared with individuals with BMI 20–<25, men and women who were obese tended to be less educated, to be physically inactive, to drink less alcohol, and not to smoke currently. Obese women also tended to use menopausal hormone therapy less.

**Table 1:** Characteristics of non-Hispanic black men and women in the NIH-AARP Diet and Health Study.

	Body Mass Index						
	15–<20	20–<25	25–<30	30–<35	35–<40	40–≤50	All
**Men** (n)	98	1,519	3,336	1,448	343	120	6864
Age at baseline (year)	62	61	61	60	60	60	61
Age at death (year)	74	74	73	73	73	72	73
College graduate (%)	35	34	34	33	27	24	33
Married (%)	66	69	73	71	71	63	71
Physical activity, ≥3 times/wk (%)	43	49	49	44	36	28	47
Alcohol, ≥30 g/day (%)	16	14	12	11	9	5	12
Never smokers (%)	32	28	29	29	29	30	29
Former smokers (%)	37	43	51	56	51	60	50
Current smokers (%)	26	22	14	10	13	7	15
**Women** (n)	206	2,048	3,639	2,219	984	511	9607
Age at baseline (year)	61	61	61	61	60	60	61
Age at death (year)	73	74	74	74	73	72	74
College graduate (%)	36	36	31	29	25	19	30
Married (%)	22	30	32	31	29	24	30
Physical activity, ≥3 times/wk (%)	37	44	42	36	30	22	39
Alcohol, ≥30 g/day (%)	10	4	3	3	3	2	3
Never smokers (%)	37	43	44	47	47	50	45
Former smokers (%)	24	34	34	35	35	35	34
Current smokers (%)	34	19	16	12	12	9	15
Menopausal hormone therapy use ever (%)	40	44	40	37	29	27	38

**Figure 1:**
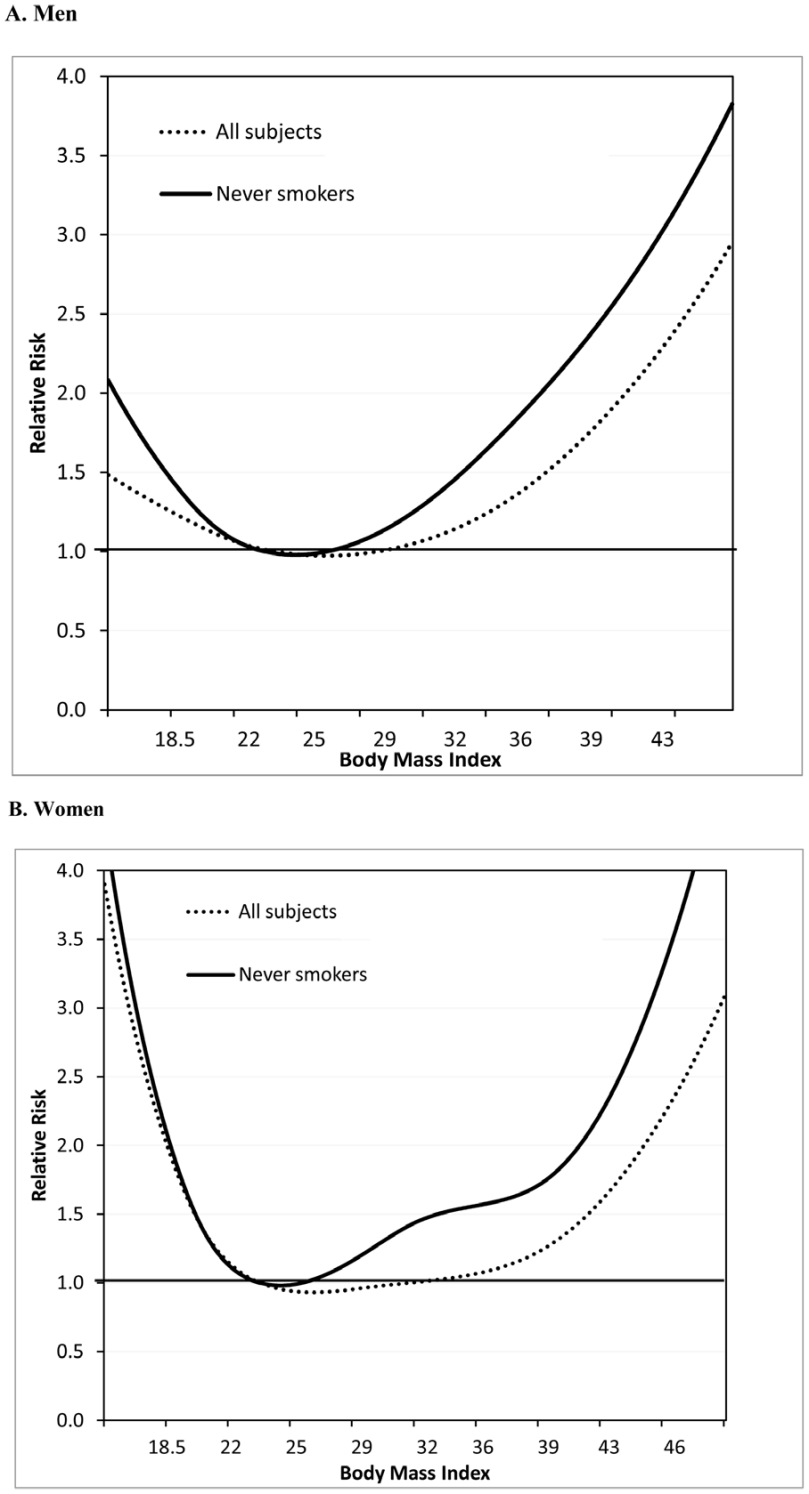
Relative risk for total mortality in non-Hispanic black men and women^a, b, c^. a. Subjects who did not have any prevalent cancer except non-melanoma skin cancer or heart disease at baseline b. Adjusted for age, education, marital status, smoking status, time since quitting smoking, number of cigarettes per day, physical activity, alcohol consumption, and menopausal hormone therapy use in women. Analysis of never smokers with no history of diseases at baseline was adjusted for same covariates except smoking status, time since quitting smoking, number of cigarettes per day. c. Men, 1,347 deaths in subjects with no history of disease and 288 deaths in never smokers with no history of diseases: Women, 1,262 deaths in subjects with no history of disease and 425 deaths in never smokers with no history of diseases.

We observed a non-linear association between BMI and total mortality in both men and women (**Figure 1**, p for nonlinearity = 0.04 in men and 0.001 in women). The risk of death gradually increased in the ranges of overweight and obese as well as in underweight. When the analysis was restricted to individuals who were never smokers, the association became more pronounced. Compared with never smokers with BMI 20–<25, the risk of death was 31% and 67% higher for those with BMI 30–<35 and BMI 35–<40, respectively, and the risk more than doubled for individuals with BMI 40–≤50 (**Table 2**). The results were similar when BMI 22.5–<25 was used as a reference group: RRs (95% CI) for BMI 30–<35, 35–<40, and 40–≤50 were 1.35 (1.05, 1.74), 1.72 (1.27, 2.32), and 2.29 (1.63, 3.21), respectively. We also observed that the results did not materially change when we excluded the first 5 years of follow-up.

**Table 2:** Relative risks (RR) and 95% confidence intervals for total mortality by categories of body mass index in non-Hispanic blacks.

	Body Mass Index						
	15–<20	20–<25	25–<30		30–<35	35–<40	40–≤50
**All subjects^a^**							
**All**							
Death (n)	74	620	985		569	226	135
Mortality rate^b^	1,986	1,324	1,086		1,238	1,382	1,912
Multivariate RR^c^	1.38 (1.08, 1.76)	1.00	0.83 (0.75, 0.92)		0.98 (0.88, 1.10)	1.13 (0.97, 1.32)	1.65 (1.36, 2.00)
**Men**							
Death (n)	23	329	585		283	91	36
Mortality rate	1,802	1,679	1,375		1,614	2,275	2,939
Multivariate RR	0.99 (0.65, 1.52)	1.00	0.89 (0.78, 1.02)		1.05 (0.90, 1.24)	1.42 (1.12, 1.79)	1.82 (1.28, 2.59)
**Women**							
Death (n)	51	291	400		286	135	99
Mortality rate	2,052	1,066	827		1,010	1,103	1,684
Multivariate RR	1.63 (1.21, 2.21)	1.00	0.77 (0.66, 0.89)		0.92 (0.78, 1.08)	0.96 (0.78, 1.18)	1.52 (1.20, 1.93)
**Never smokers**							
**All**							
Death (n)	21	127	244	179		85	57
Mortality rate	1,514	710	721	956		1,220	1,616
Multivariate RR^d^	1.97 (1.24, 3.13)	1.00	0.97 (0.78, 1.20)	1.31 (1.04, 1.65)		1.67 (1.27, 2.21)	2.23 (1.61, 3.07)
**Men**							
Death (n)	8	47	127	68		26	12
Mortality rate	1,735	797	1,037	1,307		2,219	2,914
Multivariate RR	2.12 (1.00, 4.52)	1.00	1.27 (0.91, 1.78)	1.56 (1.07, 2.28)		2.48 (1.53, 4.05)	2.80 (1.46, 5.39)
**Women**							
Death (n)	13	80	117	111		59	45
Mortality rate	1,345	668	538	824		1,019	1,424
Multivariate RR	1.85 (1.03, 3.33)	1.00	0.78 (0.59, 1.04)	1.17 (0.88, 1.57)		1.35 (0.96, 1.90)	1.93 (1.33, 2.81)

a. Subjects who did not have any prevalent cancer except non-melanoma skin cancer or heart disease at baseline.

b. Per 100,000 person-years, directly standardized to the age distribution of the cohort according to sex.

c. Adjusted for age, sex, education (less than high school, high school graduate, some college, and college graduate/postgraduate), marital status (married and not married), smoking status (never, former, current), time since quitting smoking (never, stopped ≥10 years ago, stopped 5–9 years ago, stopped 1–4 years ago, stopped <1 year ago, and currently smoking), number of cigarettes per day (0, 1–10, 11–20, 21–30, 31–40, 41–50, 51–60, and >60 cigarettes/day), physical activity (never/rare, ≤3 times/mo, and 1–2 and ≥3 times/wk), alcohol consumption (0, >0–<15, 15–<30, and ≥30 g/day) and menopausal hormone therapy use in women (never, ever). In analysis of men and women, sex was excluded from the list of covariates.

d. Adjusted for same covariates as noted in footnote c, except smoking status, time since quitting, and number of cigarettes per day. In analysis of men and women separately, sex was excluded from the list of covariates.

When men and women were examined separately, we observed significantly increased risks of death for BMI 30–<35, 35–<40, and 40–≤50 in never smoked men (RR = 1.56, 2.48, and 2.80, respectively), while the risks for the same categories of BMI were weaker in never smoked women (RR = 1.17, 1.35, and 1.93, respectively). Among never smokers, the lowest mortality rate per 100,000 person years was observed in men with BMI 20–<25 and in women with BMI 25–<30. When we narrowed the BMI categories further, the lowest mortality rate was found in men with BMI 22.5–<25 and in women with BMI 25–<27.5.

**Figure 2:**
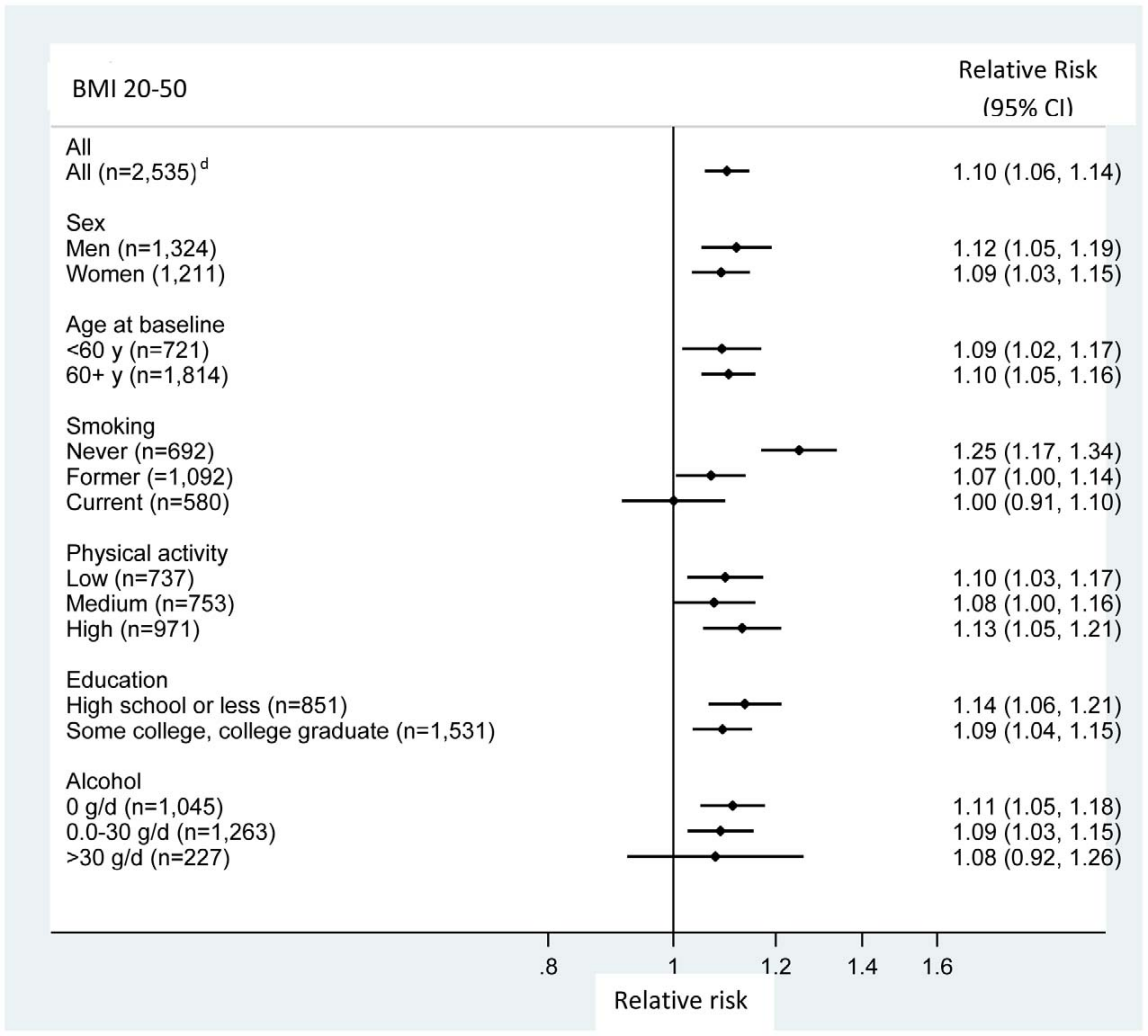
Relative risk for total mortality per 5-unit increase in body mass index in non-Hispanic blacks, according to selected characteristics^a, b, c^. a. Subjects who did not have any prevalent cancer except non-melanoma skin cancer or heart disease at baseline. b. Adjusted for following variables, except for the stratification variable in each analysis: age, sex, education, marital status, smoking status, time since quitting smoking, number of cigarettes per day, physical activity, alcohol consumption, and menopausal hormone therapy use in women. c. Markers indicate the relative risks and horizontal lines indicate 95% confidence intervals. d. Number of death; p value for interaction: 0.55 for sex, 0.50 for age at baseline, <0.001 for smoking, 0.75 for physical activity, 0.40 for education, and 0.13 for alcohol.

We also examined the association between BMI and total mortality in subgroups defined by sex, age at baseline, smoking, physical activity, education and alcohol consumption among individuals who had a BMI ≥20. We observed a positive association between BMI and total mortality persisted across these subgroups, except for smoking (**Figure 2**). A significantly positive association between BMI and total mortality was found in never smokers (RR = 1.25, 95% CI: 1.17, 1.34 for a 5-unit increase in BMI). The association was attenuated in former smokers (RR = 1.07, 95% CI: 1.00, 1.14) and was not significant in current smokers (RR = 1.00, 95% CI: 0.91, 1.10, p for interaction for smoking = <0.001).

We also examined the relation of BMI to cause-specific deaths and found significantly increased risks of death from cardiovascular diseases and from other causes in obese individuals who were never smokers (**Table 3**). BMI was not related to the risk of death from cancer.

**Table 3:** Relative risks (RR) and 95% confidence intervals for cause-specific deaths in non-Hispanic black never smokers ^a^.

	Body Mass Index				
	18–<20	20–<25	25–<30	30–<35	35–≤50
Cardiovascular disease death					
Death (n)	2	36	80	54	46
Multivariate RR^b^	0.64 (0.15, 2.66)	1.00	1.12 (0.75, 1.66)	1.46 (0.95, 2.23)	2.33 (1.48, 3.65)
Cancer death					
Death (n)	5	33	64	50	30
Multivariate RR	1.85 (0.72, 4.75)	1.00	0.97 (0.64, 1.48)	1.34 (0.86, 2.08)	1.38 (0.83, 2.29)
Other causes of death					
Death (n)	6	18	40	41	31
Multivariate RR	3.85 (1.52, 9.72)	1.00	1.10 (0.63, 1.93)	2.07 (1.18, 3.61)	2.60 (1.43, 4.72)

a. Never smokers who did not have any prevalent cancer except non-melanoma skin cancer or heart disease at baseline.

b. Adjusted for age, sex, education (less than high school, high school graduate, some college, and college graduate/postgraduate), marital status (married and not married), physical activity (never/rare, ≤3 times/mo, and 1–2 and ≥3 times/wk), alcohol consumption (0, >0–<15, 15–<30, and ≥30 g/day) and menopausal hormone therapy use in women (never, ever).

## Discussion

In this prospective cohort study following 16,471 black men and black women for an average of 13 years, we found a non-linear association between BMI and total mortality: compared with BMI between 20 and 30, BMI above and below this range were related to an increased risk of death. We also found that the association became more pronounced when the analysis was restricted to individuals who were never smokers.

A limited number of studies have examined the association between BMI and total mortality in blacks and have reported inconsistent results. Several studies suggested an increased risk of death with higher BMI [9], [14], [15], [21] while others found no association between BMI and total mortality [5], [6], [7], [8], [10], [11], [22]. Earlier studies, such as a study of black members of the Kaiser Foundation Health Plan in northern California in 1966–1973 [5] and the Charleston Heart Study [6], [7], found a J-shaped association between BMI and total mortality in black men, but no association in black women. In a few subsequent studies, however, no relation of higher BMI to total mortality was observed in either black men or black women [8], [10], [11], [22]. These studies were small and included smokers and individuals with pre-existing chronic diseases, possibly introducing bias into the results. The effect of those methodologic limitations on the association between BMI and total mortality has been reported in previous studies [3], [9], [12].

The Southern Community Cohort Study, a large cohort of low-income black men and women who were 52 years old on average, found that the risk of death was lower among overweight and obese individuals who were former or never smokers and had no history of cancer, heart disease, or stroke: compared with normal weight, RR (95% CI) for death was 0.72 (0.61–0.86) for overweight, 0.66 (0.54–0.80) for class I obesity, 0.72 (0.58–0.89) for class II obesity and 1.09 (0.89–1.35) for class III obesity [13]. In contrast, the Cancer Prevention Study II [9] which excluded ever smokers and individuals with pre-existing diseases at baseline found a non-significant positive association between obesity and total mortality in black men: RRs for BMI 28–<30, 30–<32, and ≥32, compared with BMI 23.5–<25, were 1.28, 1.29, and 1.35, respectively. Our study also found that a high BMI was related to an increased risk of death and the risk was more than doubled in black men with class II obesity (RR = 2.48) and class III obesity (RR = 2.80) compared with BMI 20–<25. Furthermore, the Multiethnic Cohort Study found that BMI was related to a significantly increased risk of death in black men who were never smokers: compared with BMI 23–<25, RR (95% CI) was 1.08 (0.78, 1.48) for BMI 25–<27.5, 1.64 (1.18, 2.28) for BMI 27.5–<30, 1.87 (1.36, 2.58) for BMI 30–<35, and 2.72 (1.81, 4.08) for BMI ≥35 [15]. Several noticeable differences in the characteristics of study participants exist between the Southern Community Cohort and other studies, including ours. The majority of Southern Community Cohort participants had income less than $15,000; 78% of black men and 52% of black women were current or former smokers; and 64% of black men and 84% of black women were overweight or obese. Interestingly, the Southern Community Cohort Study found significant interactions between income or education and BMI. The risk of death associated with obesity was lower among individuals who had income<$15,000 or had <12 years of education than among those with income ≥$15,000 or ≥12 years of education. Our study, however, did not find an interaction between education and BMI. The association of BMI with total mortality in individuals with ≤12 years of education was similar to that of those with >12 years of education in our study.

In black women who were never smokers and had no cancer or heart disease at baseline, class II and III obesity, but not overweight and class I obesity, were related to an increased risk of death in our study and in the Cancer Prevention Study II [9]. Conversely, the Black Women’s Health Study reported a significantly increased total mortality among overweight (RR = 1.31, 95% CI:1.01, 1.72, BMI 27.5–<30 vs. 22.5–<25) and class I (RR = 1.27, 95% CI:0.99, 1.64), class II (RR = 1.51, 95% CI:1.13, 2.02), and class III (RR = 2.19, 95% CI:1.62, 2.95) obese women who were healthy never smokers [14]. The Multiethnic Cohort Study also found an increased risk of total mortality among never smoked black women: RR (95% CI) was 1.23 (0.97, 1.54) for BMI 27.5–<30, 1.45 (1.16, 1.80) for BMI 30–<35, and 1.74 (1.37, 2.20) for BMI ≥35 compared with BMI 23.0–<25. In addition, the Women’s Health Initiative Observational Study [21] reported a significantly increased total mortality with class I (RR = 1.35, 95% C: 1.01, 1.80 vs. BMI 18.5–<25), class II (RR = 1.99, 95% CI:1.44, 2.73), and class III obesity (RR = 1.55, 95% CI:1.07, 2.25) in healthy black women, but no association between overweight and total mortality. A stronger association of overweight with total mortality in the Black Women’s Health Study as compared to our study may be partially explained by the age of the study population. Most of the Black Women’s Health Study participants were 30–50 years old while our study participants were 50–71 years old. In the analysis of the older population, the possibility of residual confounding by undiagnosed diseases related to weight loss may be higher than in the younger population, which may attenuate the association between BMI and total mortality. Previous studies of whites found a stronger relation of BMI to total mortality in younger populations than in older populations [2], [3].

Our finding of a positive association between BMI and total mortality in black men is consistent with the findings in whites: the RR of 1.56, 2.48 and 2.80 for class I, II and III obesity, respectively, found in black never smokers in our study were very similar to corresponding RRs of 1.44, 2.06, and 2.93 observed in the pooled analysis of 1.46 million non-Hispanic white men [3]. Also, the lowest mortality rate was found among black men with BMI 22.5–<25 in our study and white men in the pooled analysis [3].

On the other hand, the relation of BMI to total mortality in black women somewhat differed from that of white women. In contrast to an increased risk of death among overweight white women reported in the large pooled analysis [3], our study as well as other studies [8], [9], [10], [11] except for the Black Women’s Healthy Study, did not find that overweight was associated with the total mortality in black women. In addition, BMI for the lowest mortality rate in black women in our study (BMI 25–<27.5) was higher than that of white women (BMI 22.5–<25) [3]. Similarly other studies showed that BMI for the lowest mortality rate in black women was higher than that of white women [6], [8], [9], [11]. Another study [23] also found that the optimal BMI threshold for cardiometabolic risk was 3 units of BMI higher in black women than in white women, but no difference was found between black men and white men. Furthermore, studies with direct measures of body fat reported that black women had smaller amount of visceral adipose tissue for a given BMI than white women [24], [25]. This racial difference in the amount of body fat may partially explain the lack of association between overweight and total mortality in black women in our and other studies. Future studies are needed to investigate whether the different results between black women and white women are due to biologic differences or to methodologic limitations.

The strengths of our study include the relatively large population size and a wide range of BMI that enabled us to examine the association with class II and III obesity. We also conducted analyses by excluding individuals with a history of cancer or heart disease at baseline and/or smokers who tended to have lower BMI but have higher risk of death. As previous studies have shown [3], [9], [12], inclusion of individuals with preexisting diseases or smokers attenuated the association between BMI and total mortality, and adjusting for smoking in a multivariate analysis may not be adequate to control for confounding. Furthermore, we excluded individuals with less than 1 year of follow-up to reduce the effect of reverse causation and used BMI 20–<25, rather than BMI 18.5–<25, as a reference group because BMI 18.5–<20 may still include individuals with undiagnosed chronic diseases.

Our study also has several limitations. BMI used in the study was estimated based on self-reported height and body weight at baseline. Self-reported BMI, however, has shown high correlation (r = 0.93) with measured BMI in blacks in the National Health and Nutrition Examination Study III [26]. Also, both self-reported and measured BMI were similarly correlated with biomarkers of chronic diseases, suggesting that self-reported BMI provides sufficient accuracy for epidemiologic studies. Information on body weight and history of diseases were also collected only once at baseline, therefore our analysis could not address any changes which occurred during follow-up. Moreover, other measures of body adiposity such as waist circumference and waist and hip ratio were not investigated in the current study. Even though we excluded individuals with history of cancer and heart disease at baseline, we cannot rule out the possibility that undiagnosed diseases may have attenuated the association between BMI and total mortality. Lastly, although our study was larger than previous studies, we still have limited power to examine the association in subgroups (e.g. age, duration of follow-up) or associations with cause-specific deaths among individuals who were never smokers and had no history of diseases at baseline.

We recognize that other existing studies of BMI and total mortality in blacks also had similar limitations due to small sample size. Because previous studies used a different reference group and different exclusion criteria, it is neither feasible to directly compare one study result to another nor to conduct a meta-analysis. Given the public health importance of this topic and to overcome the limitations in existing studies, a pooled analysis of comparable studies is warranted.

In conclusion, we found that obesity was associated with an increased risk of death in blacks. However, the association in black women was weaker than that of white women in previously published studies. Future studies with larger samples or a pooled analysis of existing studies, capable of systematically evaluating the association between a wide range of BMI and total mortality in various subgroups, are warranted.
